# *Astragalus membranaceus* (Huangqi) and *Rhizoma curcumae* (Ezhu) decoction suppresses colorectal cancer via downregulation of Wnt5/β-Catenin signal

**DOI:** 10.1186/s13020-021-00564-6

**Published:** 2022-01-06

**Authors:** Yong Bian, Gang Wang, Jing Zhou, Gang Yin, Tiantian Liu, Li Liang, Xinyue Yang, Wen Zhang, Kexin Ni, Decai Tang, Yun Yu

**Affiliations:** 1 Laboratory Animal Center, Nanjing University of Chinese Medicine, Nanjing, 210023 China; 2 Center of Experimental Animals, Sichuan Academy of Chinese Medicine Sciences, Chengdu, 610041 China; 3 School of Traditional Chinese Medicine and School of Integrated Chinese and Western Medicine, Nanjing University of Chinese Medicine, Nanjing, 210023 China; 4 Jiangsu Key Laboratory of Oral Diseases, Nanjing Medical University, Nanjing, 211166 China; 5 School of Pharmacy, Nanjing University of Chinese Medicine, Nanjing, 210023 China

**Keywords:** *Astragalus membranaceus*, *Rhizoma curcumae*, Wnt5, β-Catenin, Colorectal cancer

## Abstract

**Background:**

The decoction of *Astragalus membranaceus* (Huangqi) and *Rhizoma curcumae* (Ezhu) has been reported as a potential antitumor agent for colorectal cancer (CRC) in experimental and clinical studies, but its underlying mechanism is still unclear.

**Methods:**

The current research aims to explore the potential of *Astragalus membranaceus* (Huangqi) and *Rhizoma curcumae* (Ezhu) decoction (AR decoction) in the treatment of CRC and explore the underlying mechanism. SW620 cells were transient transfection to overexpress or knock down wnt 5 or β-Catenin. *Astragalus membranaceus* (Huangqi) and *Rhizoma curcumae* (Ezhu) -containing serum (AR-CS) was used to interfere with SW620 cells. Additional AR-CS, Wnt5 inhibitor (IWP-4), and β-Catenin inhibitor (JW55) were used to intervene in SW620 cells. Furthermore, subcutaneously injection of SW620 cells into the right flank of nude mice replicated xenograft mice, which were treated with AR decoction for 21 days.

**Results:**

AR-CS significantly reduced the mRNA and protein expression levels of Wnt5, β-Catenin, ARF6, and N-Cadherin in SW620 cells, while inhibiting the proliferation and migration of SW620 cells. In cells overexpressing Wnt5 or β-Catenin, these effects of AR-CS were significantly suppressed. On the contrary, the inhibitory effect of AR-CS on the mRNA and protein levels of ARF6 and N-Cadherin and cell proliferation and migration of SW620 was enhanced, when Wnt5 or β-Catenin were knocked down or suppressed by the inhibitors. Moreover, in the mouse model of xenograft tumors, AR decoction not only reduced the tumor volume and inhibited the mRNA levels and protein levels of Wnt5, β-Catenin, ARF6, and N-Cadherin in the tumor, but also inhibit the protein levels of LRP5, LRP6, TCF-4, and LEF1.The histopathology of mice also showed increased apoptosis in tumor tissues, and AR decoction treatment did not cause pathological damage to the kidney and liver.

**Conclusions:**

Our results provide evidence that AR decoction inhibits Wnt5/β-catenin signaling and inhibits the development of CRC, which is a promising traditional medicine in the clinical treatment of CRC.

## Introduction

Colorectal cancer (CRC), including colon cancer and rectal cancer, is the most common human digestive tract malignancy. In 2015, the incidence and mortality of CRC ranked fifth among all malignant tumors in China [1]. According to global cancer statistics in 2018, colon cancer will have become the world's third most common cancer and the second cause of tumor-related death [2, 3].

In China, traditional Chinese medicine combined with chemotherapy is part of the most important methods for the comprehensive treatment of colorectal cancer [4]. More importantly, many traditional Chinese medicine exhibit potential anticancer effect [5], including the colorectal cancer [6]. Among these Chinese herbs, it has been clear that *Astragalus membranaceus* (Huangqi) [7] and *Rhizoma curcumae* (Ezhu) [8] have antitumor effects in a variety of tumors, including lung Cancer [9, 10] or breast cancer [11, 12] etc. In clinical practice in China, *Astragalus membranaceus* (Huangqi) or *Rhizoma curcumae* (Ezhu) have been used for cancer patients, and the survival and quality of life of patients with colorectal cancer was significantly improved after treatment [13]. The decoction of *Astragalus membranaceus* (Huangqi) or *Rhizoma curcumae* (Ezhu) has also shown its potential in the treatment of colorectal cancer in experimental studies [14, 15], but its underlying mechanism is still unclear. So, the therapeutic effect of *Astragalus membranaceus* (Huangqi) in combination with *Rhizoma curcumae* (Ezhu) on colorectal cancer needs to be confirmed in more studies.

Disorders of the Wnt/β-catenin signaling pathway are widely present in different types of malignant tumors, and are particularly significant in digestive system tumors [16]. In colorectal cancer, about 80% of patients have abnormal activation of the Wnt pathway [17]. The Wnt/β-Catenin signaling pathway promotes the abnormal proliferation of intestinal epithelial cells, the disappearance of normal intestinal wall structure, and ultimately leads to the occurrence of CRC [18, 19]. Hyperactivation of Wnt signal promotes the transfer of β-catenin from the cytoplasm to the nucleus and accelerates the process of transforming benign colon tumors to malignant tumors [20]. The Wnt/β-catenin signaling pathway and its related factors have become diagnostic and prognostic indicators of gastrointestinal tumors [21, 22]. Therefore, Targeting Wnt/β-catenin to treat CRC has emerged as a therapeutic strategy and been continuously concerned by researchers [23].

In this study, we explored the potential of *Astragalus membranaceus* (Huangqi) in combination with *Rhizoma curcumae* (Ezhu) decoction (AR decoction) in the treatment of CRC. First, the rat *Astragalus membranaceus* (Huangqi) and *Rhizoma curcumae* (Ezhu)–containing serum (AR-CS) was used in vitro to evaluate the inhibitory effects of *Astragalus membranaceus* (Huangqi) and *Rhizoma curcumae* (Ezhu) on the proliferation and migration of colorectal cancer cells. Further, we provided the evidence for its treatment of CRC by inhibiting Wnt/β-catenin signaling in vitro. Importantly, AR decoction could inhibit tumor growth and inhibit Wnt/β-catenin signaling in mouse models of CRC xenograft tumors.

## Materials and methods

### Reagents

IWP-4 (#HY-12879) and JW55 (#HY-13968) were purchased from MedChemExpress (New Jersey, USA). CCK8 kit (#CA1210), 4% paraformaldehyde (#P1110), hematoxylin–eosin staining kit (#G1120), Trizol(#R1100), BCA Protein Quantitative Kit (#PC0020), and 30% acrylamide (29:1) (#A1010) were purchased from Solarbio life sciences (Beijing, China). HiFiScript gDNA Removal RT MasterMix (#CW2020) and SuperRT One Step RT-PCR Kit (#CW0742) were purchased from cwbiosciences (Jiangsu, China). Protein loading buffer (#P0015L), protein pre-staining Marker(#P0069), and BeyoECL Star Luminescent Liquid (#P0018AS) were purchased from Beyotime (Shanghai, China). Cr(C011-2-1), BUN (C013-2-1), AST (C010-2-1), and ALT(C009-2–1), detection kits were purchased from Nanjing Jiancheng Bioengineering Institue (Nanjing, Chian). Antibody for Wnt5 (#55184-1-AP), β-Catenin (#51067-2-AP), ARF6 (#20225-1-AP), and N-Cadherin (#22018-1-AP), and GAPDH (#60004-1-Ig), HRP-conjugated Affinipure Goat Anti-Rabbit IgG(H + L) (#SA00001-2), and FITC-conjugated Affinipure Goat Anti-Rabbit IgG(H + L) (#SA00003-2) were purchased from Proteintech (Wuhan, China). Antibody for LRP5 (ab223203), LRP6 (ab134146), TCF-4 (ab130014), and LEF1 (ab137872) were purchased from abcam (Cambridge, UK).

### Drugs and drug-containing serum preparation

*Astragalus membranaceus* (Huangqi) and *Rhizoma curcumae* (Ezhu) were purchased from China Traditional Chinese Medicine Co., Ltd. AR decoction are obtained by soaking the traditional Chinese medicine *Astragalus membranaceus* (Huangqi) and *Rhizoma* (Ezhu) *curcumae* decoction with traditional water decoction [24]. *Astragalus membranaceus* (Huangqi) (500 g) and *Rhizoma curcumae* (Ezhu) (500 g) were weighed and added to a stainless pot. After 2 l water added and boiled for 1 h, the liquid was collected. Then 2 l water is added and boiled for another 1 h to collect liquid again. The solution that was combined twice, a rotary evaporator was used to concentrate the solution and the final volume was concentrated to 500 ml. The concentration of *Astragalus membranaceus* (Huangqi) and *Rhizoma curcumae* (Ezhu) is (1 g *Astragalus membranaceus* (Huangqi) + 1 g *Rhizoma curcumae* (Ezhu))/ml. The clinical dosage of *Astragalus membranaceus* and *Rhizoma curcumae* is (25 g + 25 g)/day. Based on the conversion method drove from body surface coefficient of mice (Body surface area ratio of mice and humans = 0.0026) or rat (Body surface area ratio of rat and humans = 0.018) and human. The dose for mice is 6.5 g/kg, the dose for rats is 4.5 g/kg. They were calculated as follows: Mice: 50 g/day (human dosage)*0.0026/0.02 kg(weight of mice) = 6.5 g/kg/day. Rat: 50 g/day (human dosage)*0.018/0.2 kg(weight of rat) = 4.5 g/kg/day.

All procedures of animals described in this study were performed in accordance to the guidelines of the Institutional Animal Ethical Committee of Nanjing University of Chinese Medicine and in accordance with the ethical standards established by Ethics Committee of Nanjing University of Chinese Medicine. Forty male SD rats with SPF grade, 12 weeks old, weighing 250–280 g, were housed in a standard SPF animal feeding center (with temperature 20–26 ℃, humidity 40–70%, and 12-h day/night light cycle). After a week of adaptation, 20 of the rats were intragastric administration with AR decoction (4.5 g/kg) for seven consecutive days. Other 20 rats were intragastric administration with water. After seven days, these rats were deeply anesthetized and blood was taken from the common intracranial artery into the pro-coagulation tube [25]. Each rat could obtain about 10 ml of whole blood. After centrifugation (3000 rpm, 10 min), the serum of the rats in the same group was collected and mixed. At least 100 ml AR-CS and 100 ml normal rat serum were obtained.

### Quality control of drugs and drug-containing serum

The content of representative substances in AR decoction and AR-CS was determined by Agilent 1260 high performance liquid chromatograph HPLC (Agilent, USA). The chromatographic column is a Hedera ODS-2 C18 chromatographic column (4.6 mm × 250 mm, 5 μm, Jiangsu Hanbang Technology Co., Ltd.). Methanol-0.1% phosphoric acid aqueous solution (A: B) was used as mobile phase, gradient elution, elution time and mobile phase ratio are shown in Table 1. Flow rate was 1.0 ml/min, injection volume was 20 μl, column temperature was 30 °C, Full-wavelength scanning, extraction wavelengths were 215 and 420 nm.

**Table 1:** Methanol-0.1% phosphoric acid aqueous solution mobile phase elution time and solution ratio

Time	A (V/V %)	B (V/V %)
0	10	90
4	20	80
6	25	75
15	30	70
30	40	60
50	70	30
70	70	30

Sample pretreatment: For AR decoction, 1 ml of extract was accurately drawn and add 1 ml of absolute ethanol. After mixing, it was standed for 24 h, centrifuged at 13,000 rpm for 10 min. Then the supernatant was taken, and passed through 0.22 μm filter membrane. And the sample was injected.

For AR-CS: 400 μl of serum was precisely aspirated, precisely added 1.6 ml of methanol, extracted with ultrasound for 10 min, centrifuged at 13,000 rpm for 10 min. Then 1.8 ml of the supernatant was precisely aspirated, blowed dry with N2, and added 100 μl of methanol-0.1% phosphoric acid aqueous solution (20:80) for redissolve, centrifuged at 13,000 rpm for 10 min, and the supernatant was taken for injection.

**Fig. 1:**
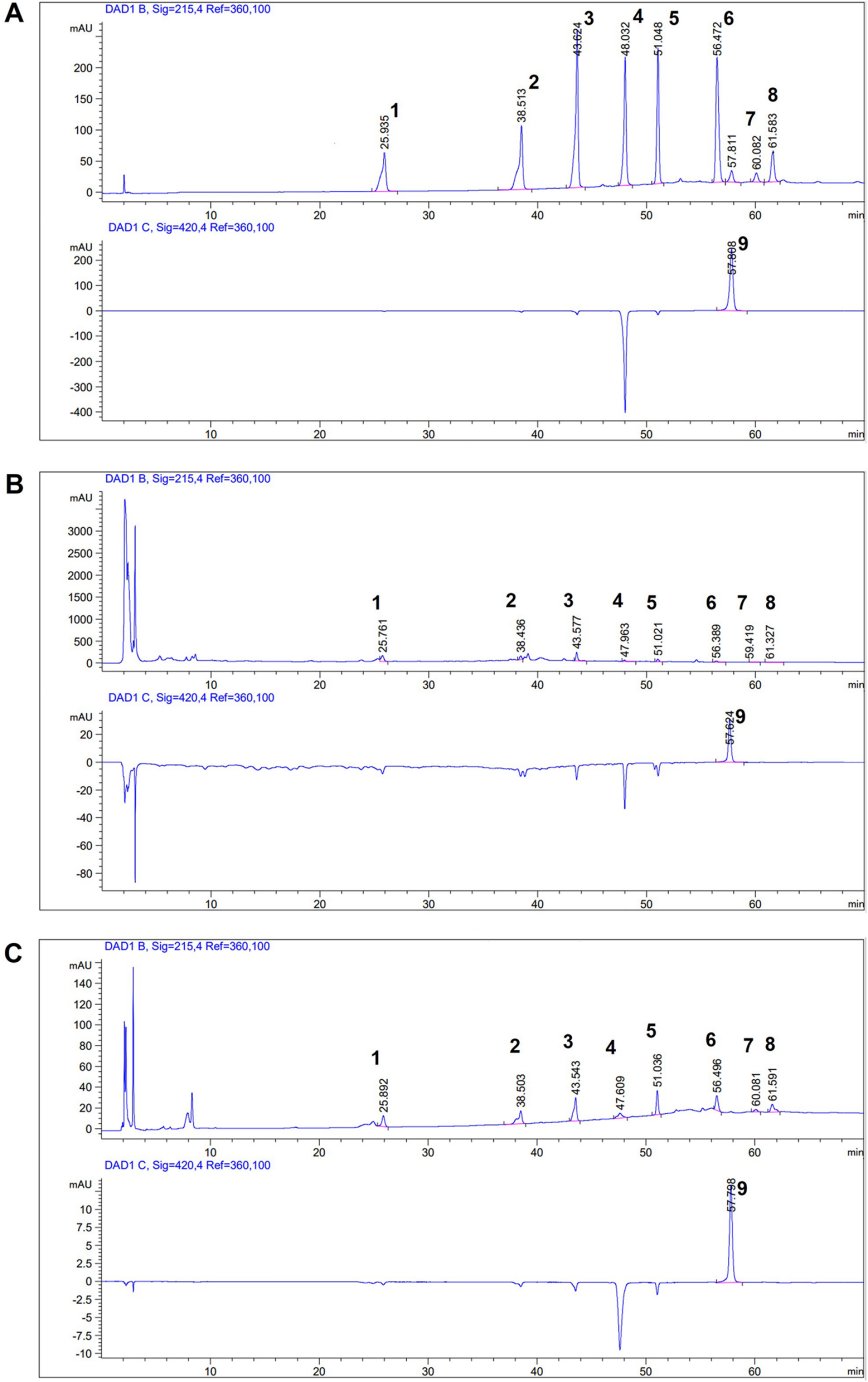
Representative HPLC chromatograms of AR decoction and AR-CS. Representative HPLC chromatograms of standard substance mixture (**A**), AR decoction (**B**), and AR-CS (**C**) showed retention peaks of nine substances. They were 1: Calycosin-7-glucoside, 2: Ononin, 3: Calycosin, 4: Kaempferol, 5: Formononetin, 6: Curdion, 7: Curzerene, 8: Isocurcumenol, and 9 curcumin

Nine main components in AR extract were quantitatively detected by HPLC, among which the maximum absorption wavelength of Calycosin-7-glucoside (CAS: 20633-67-4), Ononin (CAS: 486-62-4), Calycosin (CAS: 20575-57-9), Kaempferol (CAS: 520-18-3), Formononetin (CAS: 485-72-3), Curdion (CAS: 13657-68-6), Curzerene (CAS: 17910-09-7), and Isocurcumenol (CAS: 24063-71-6) were 215 nm. The contents in the extract were 0.08, 0.05, 0.04, 0.03, 0.02, 0.03, 0.02 mg/ml, and the contents in the serum of rats were 0.79, 2.09, 1.01, 1.26, 1.07, 2.06, 0.77, 0.81 μg/ml, respectively. The maximum absorption wavelength of curcumin was 420 nm, and the content of curcumin in extract and serum after administration were 0.01 mg/ml and 0.68 μg/ml, respectively. The results are shown in Fig. 1 and Table 2. All the standard substances (purity > 98%) were purchased from National Institute of Metrology (Beijing, China).

**Table 2:** Content of 9 compounds in AR decoction and AR-CS

Name	Wave (nm)	Standard solution		AR decoction		AR-CS	
		RT	Content	RT	Content	RT	Content
		min	mg/ml	min	mg/ml	min	μg/ml
Calycosin-7-glucoside	215	25.935	0.04	25.761	0.08	25.892	0.79
Ononin	215	38.513	0.06	38.436	0.05	38.503	2.09
Calycosin	215	43.624	0.04	43.577	0.04	43.543	1.01
Kaempferol	215	48.032	0.11	47.963	0.03	47.609	1.26
Formononetin	215	51.048	0.04	51.021	0.02	51.036	1.07
Curdion	215	56.472	0.12	56.389	0.03	56.496	2.06
Curzerene	215	60.082	0.02	60.019	0.02	60.081	0.77
d Isocurcumenol	215	61.583	0.03	61.327	0.02	61.591	0.81
curcumin	420	57.808	0.05	57.624	0.01	57.798	0.68

RT, retention time

### Cell culture and transient transfection

SW620 cell line was purchased from ATCC. SW620 cells were cultured with DMEM (#06-1170-87-1A, Biological Industries, Israel) containing 10% fetal bovine serum (#04-011-1A, Biological Industries, Israel), 100 U/ml penicillin and 100 mg/mL streptomycin (#03-034-1B, Biological Industries, Israel). SW620 cells were incubated in a cell incubator (Thermo Scientific, USA) containing 5% CO_2_, at 37 °C.

Wnt 5 (#sc-41112) and β-catenin (#sc-29209) siRNA and control (#sc-37007) siRNA were purchased from Santa Cruz Biotechnology (CA, USA). pcDNA-Wnt 5 and pcDNA-β-catenin and control vector were purchased from Addgene (Cambridge, UK). SW620 cells were cultured in DMEM medium at a density of 10^5^ cells in 6-well plates. According to the manufacturer's instructions, lipofectamine 3000 reagent (#L3000015, Invitrogen, CA, USA), pcDNA-Wnt 5 (2 μg), pcDNA-β-catenin (2 μg), control vector (2 μg) or wnt 5 siRNA, β-catenin siRNA (50 nM) and control siRNA (50 nM) were used to transiently transfect into cells. After 6 h of transfection, complete culture medium was added to the transfection medium, continue culturing for 12 h. Then the cells are collected for inoculation.

### CCK-8 assay

Cell Count Kit-8 (CCK-8) was used to test cell viability. The current research conducted multiple cell viability tests, including Sw620 cells were treated with different concentrations of AR solution for 12 h, and SW620 cells were treated with different concentrations of FBS or rat serum for 12 h. Different concentrations of AR-CS (0%, 20%, 40%, 80%, 100%) were used to incubate SW620 cells for 12 h, or 20% AR-CS treated SW620 cells for different administration time (0 h, 3 h, 6 h, 9 h, 12 h, 18 h, 24 h) were another two independent experiments. When different concentrations of AR-CS (0%, 20%, 10%, 5%) were used on SW620 cells for 24 h, diffferent concentrations of blank rat serum(20%, 0%, 10%, 15%) were added to make the serum concentration to 20% in all groups.

For cell viability detection, SW620 cells were seeded at a density of 5000 cells/well in 96-well plates. After the cells adhered, different concentrations of drug-containing serum were added to SW620 cells for different time. 10 μl of CCK-8 reagent was added to the 96-well plate 2 h before the termination of the experiment. After two hours, microplate reader was used to determine the absorbance (OD) value at a wavelength of 450 nm. The cell viability of the control group was defined as 100%, and the cell viability was calculated according to the formula: Cell viability = (OD of test group/Everage OD of control group)*l00%.

### Transwell assay

The current study performed four Transwell assays. The treatment of SW620 cells in these four cell experiments is different. In the first experiment, different concentrations of AR-CS (0%, 20%, 10%, 5%) treated SW620 cells for 24 h. In the second experiment, WT, Wnt5 over-expressed, and β-Catenin over-expressed SW620 cells with or without 20% AR-CS treated for 12 h. In the third experiment, WT, Wnt5 knock-down, and β-Catenin knock-down SW620 cells with or without 20% AR-CS treated for 24 h. In the fourth test, SW620 cells were treated with or without 20% AR-CS, Wnt5 inhibitor (IWP-4), and β-Catenin inhibitor (JW55) treated for 24 h.

Briefly, WT, Wnt5 over-expressed, β-Catenin over-expressed, Wnt5 knock-down, or β-Catenin knock-down cells are digested and seeded into the upper chamber of the Transwell chamber. After adherent, cells were incubated with AR-CS and tool drugs for the corresponding time. Subsequently, the cells on the upper chamber were fixed and stained with crystal violet. The cells inside the inner membrane of the chamber were erased, and the number of cells on the underside of the membrane placed on the glass slide was observed with an inverted optical microscope. Then the pictures were taken. The cell migration rate of the normal group was defined as 100%, and the overall migration rate of the other groups was calculated. The calculation formula for the migration rate is: migration rate = (number of test group/Average number of control group)*l00%.

### Wound scratch assay

Like the Transwell experiment, the wound scratch assay also tested the wound healing rate under four different conditions. Briefly, WT, Wnt5 over-expressed, β-Catenin over-expressed, Wnt5 knock-down, or β-Catenin knock-down cells were seeded into 24-well plates after digestion. After the cells adhere to the wall, a scratch needle was used to slightly scratch along the blank center axis. After cleaning once, pictures were taken by an inverted optical microscope, and the scratch area (Area 1) was calculated. The cells were incubated with AR-CS and tool drugs for the corresponding time. Then the cells were fixed and washed. The healing of the scratches was observed under an inverted optical microscope, and the area of the scratches (Area 2) was calculated. The wound healing rate is calculated by the following formula: wound healing rate = (Area 1 − Area2)/Area1*100%.

### Immunocytofluorescent staining

Briefly, WT, Wnt5 over-expressed, β-Catenin over-expressed, Wnt5 knock-down, or β-Catenin knock-down cells were seeded into 24-well plates with glass coverslips. After the cells adhere to the coverslips, different concentrations of drug-containing serum were added to SW620 cells for different time. After that, the cells were fixed with 4% paraformaldehyde. PBS was used to clean and β-Catenin (1:100) was used to incubate the cells for overnight. FITC-conjugated goat anti-rabbit IgG (1:100) was used to incubate the cells for 1 h, and then DAPI was used to stain the cells for 5 min. Photographs were taken with a fluorescence microscope after glycerin gelatin was used to seal the coverslips on glass slides.

### Real time qPCR

The treatment of cells was the same as in the Transwell assay. A total of four different cell treatments were carried out. For collected SW620 cells or tumor tissues derived from xenograft mice, after using Trzol reagent to extract total RNA, the mRNA of SW620 cells or tumor tissues was reverse transcribed into cDNA, referring to the instruction. After referring to the PCR reaction kit instructions, the mRNA expression levels of Wnt5, β-Catenin, ARF6, and N-Cadherin were measured by the ABI7500 system. GAPDH(#B661104) primer was purchased from Sangon Biotech (Shanghai, China). The other primers were synthesized by Sangon Biotech (Shanghai, China), the sequences were as follows:

Wnt5: forward: CCTGAAGGAGAAGTACGACAG, reverse: GATGTAGACCAGGTCTTGTGTG, product size: 112 bp.

β-Catenin: forward: TGGATTGATTCGAAATCTTGCC, reverse: GAACAAGCAACTGAACTAGTCG, product size: 92 bp.

ARF6: forward: GCTCTGGCGGCATTACTACACTG,reverse: AGGATTATGGCGTCCCTCATCTCC, product size: 138 bp.

N-Cadherin: forward: CGATAAGGATCAACCCCATACA, reverse: TTCAAAGTCGATTGGTTTGACC, product size: 142 bp.

The 2^−ΔΔ^ method was used to calculate the relative expression levels of Wnt5, β-Catenin, ARF6, and N-Cadherin mRNA in SW620 cells or tumor tissues.

### Western blot

The treatment of cells was the same as in the Transwell assay. A total of four different cell treatments were carried out. For the collected SW620 cells or tumor tissues derived from xenograft mice, after using the REPI lysate to extract the total protein, the total protein level of SW620 cells or tumor tissues was determined, referring to the BCA protein determination kit instruction. After each sample was boiled with the loading buffer, the protein was separated by SDS-PAGE gel electrophoresis. After transferring the protein in the gel to the PVDF membrane, the PVDF membrane was incubated with Wnt5, β-Catenin, ARF6, N-Cadherin, LRP5, LRP6, TCF-4, and LEF1 and GAPDH overnight at 4 °C. After that, the PVDF membrane was incubated with HRP-modified goat anti-rabbit Igg for 2 h. After washing, the high-sensitivity chemiluminescence solution combined with the gel imaging system was used to visualize the blots of Wnt5, β-Catenin, ARF6, N-Cadherin, LRP5, LRP6, TCF-4, LEF1, and GAPDH in SW620 cells or tumor tissues.

### Xenograft tumor models

Fifteen nude mice with SPF grade were 6 weeks old, weighing 18–22 g. And 2 × 10^6^ SW620 cells were subcutaneously injected into the right flank of nude mice. After the cells were injected. The animals were divided into three groups: the model group, the AR treatment group and the oxaliplatin treatment group, each group contained 5 mice. AR decoction (6.5 mg/kg) was given by gavage daily for 21 days. Oxaliplatin (1 mg/kg) was used as a positive drug and was administered by intrapulmonary injection every day for 21 consecutive days. As a model control, mice were given water by gavage every day. The mice are weighed every 2 days. The length and width of the tumor were measured every three days and the volume of the tumor was calculated as length × width^2^. After 21 days, the mice were sacrificed and the tumor tissues were taken out, and the tumors were photographed and weighed.

### Hematoxylin–eosin (HE) staining

After the mice were treated, they were sacrificed and tumor tissues were obtained. The tumor mass of each mouse was incised from the middle. Half was used for the above-mentioned WB experiment, and the other half was immersed in paraformaldehyde. At the same time, the liver and kidney of mice were fixed in paraformaldehyde. Tumors, livers and kidneys from mice were coated into wax blocks after routine dehydration, transparency and wax immersion treatments. Then HE staining was performed and photographed by an ordinary light microscope. The experimental procedures of tissue embedding, sectioning and HE staining refer to the previous report [26].

### Blood biochemical test

After the mice were treated, they were sacrificed and serum was obtained. The levels of Cr, BUN, AST, and ALT in serum were detected according to the kit manufacturer's instructions.

### Statistical analysis

Statistical analysis was carried out using ANOVA. Results are expressed as mean ± SD. p < 0.05 was considered as a significant difference. All analyses were performed with GraphPad Prism Version 5.01 (GraphPad Software Inc., San Diego, CA, USA).

## Results

### AR-CS inhibits the proliferation and migration of SW620 cells

**Fig. 2:**
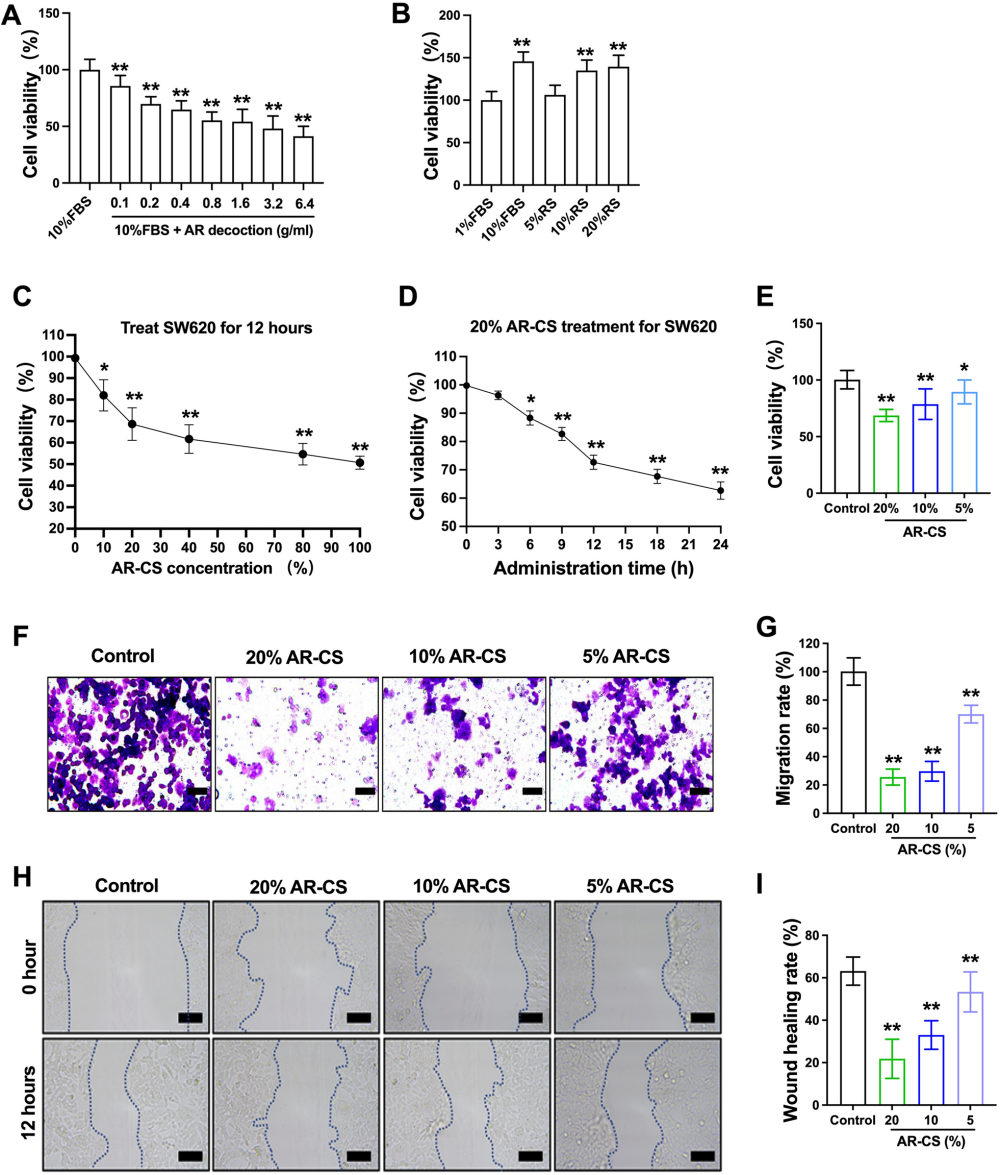
AR-CS inhibits the proliferation and migration of SW620 cells. Different concentrations of AR decoction (0.1, 0.2, 0.4, 0.8, 1.6, 3.2, 6.4 g/ml) treated SW620 cells for 12 h, CCK-8 assay was used to detect cell viability (**A**). Different concentrations of FBS (1%, 10%) or rat serum (RS) (5%, 10%, 20%) treated SW620 cells for 12 h, CCK-8 assay was used to detect cell viability (**B**). Different concentrations of AR-CS (0%, 20%, 40%, 80%, 100%) treated SW620 cells for 12 h, CCK-8 assay was used to detect cell viability (**C**). 20% AR-CS treated SW620 cells for 24 h, CCK-8 assay was used to detect cell viability in different administration time (0 h, 3 h, 6 h, 9 h, 12 h, 18 h, 24 h) (**D**). Different concentrations of AR-CS (0%, 20%, 10%, 5%) treated SW620 cells for 24 h, CCK-8 assay was used to detect cell viability (**E**), transwell assay (**F**) was used to detect cell migration rate (**G**), wound scratch assay (**H**) was used to detect cell wound healing rate (**I**). Scale bar = 100 µm in **F**, Scale bar = 400 µm in **H**. N = 10 in **a–c**, compared with 0% AR-CS treated group (control), *p < 0.05, **p < 0.01. N = 6 in **d–g,** compared with control group, **p < 0.01

Different concentrations of AR decoction (0.1, 0.2, 0.4, 0.8, 1.6, 3.2, 6.4 g/ml) were used to incubate SW620 cells for 12 h, the cell viability results showed that AR decoction (0.1–6.4 g/ml) had a significant inhibitory effect in SW620 cells cultured with 10%FBS- containing medium (Fig. 2A). Different concentrations of FBS (1%, 10%) or rat serum (RS) (5%, 10%, 20%) treatment showed that the serum concentration had significant positive-going impact on the cell viability in SW620 cells (Fig. 2A). In order to exclude the influence of serum on experimental results, blank rat serum was used in all subsequent experiments to adjust the serum concentration of 20% in all group.

SW620 cells were treated with increasing concentrations of AR-CS (0%, 20%, 40%, 80%, 100%) for 12 h, and the cell viability was detected. After 20% AR-CS was applied to SW620 cells for 12 h, the cell viability was approximately 60–70% (Fig. 2C). Then 20% of AR-CS was used on SW620 cells for different time (0 h, 3 h, 6 h, 9 h, 12 h, 18 h, 24 h). With the increase of the time, the cell activity continued to decrease, and the cell activity was about 70% after 12 h of treatment, about 60% after 24 h (Fig. 2D). A 20% / 10% / 5% gradient concentration of AR-CS was selected to act on SW620 cells for 24 h to observe cell viability again. The results showed that AR-CS concentration-dependently reduced the viability of SW620 cells (Fig. 2E). Transwell assay and wound scratch assay showed that cell migration rate and wound healing rate were also reduced in a concentration-dependent manner by AR-CS (Fig. 2F–I).

### AR-CS inhibits Wnt/β-Catenin signaling of SW620 cells

**Fig. 3:**
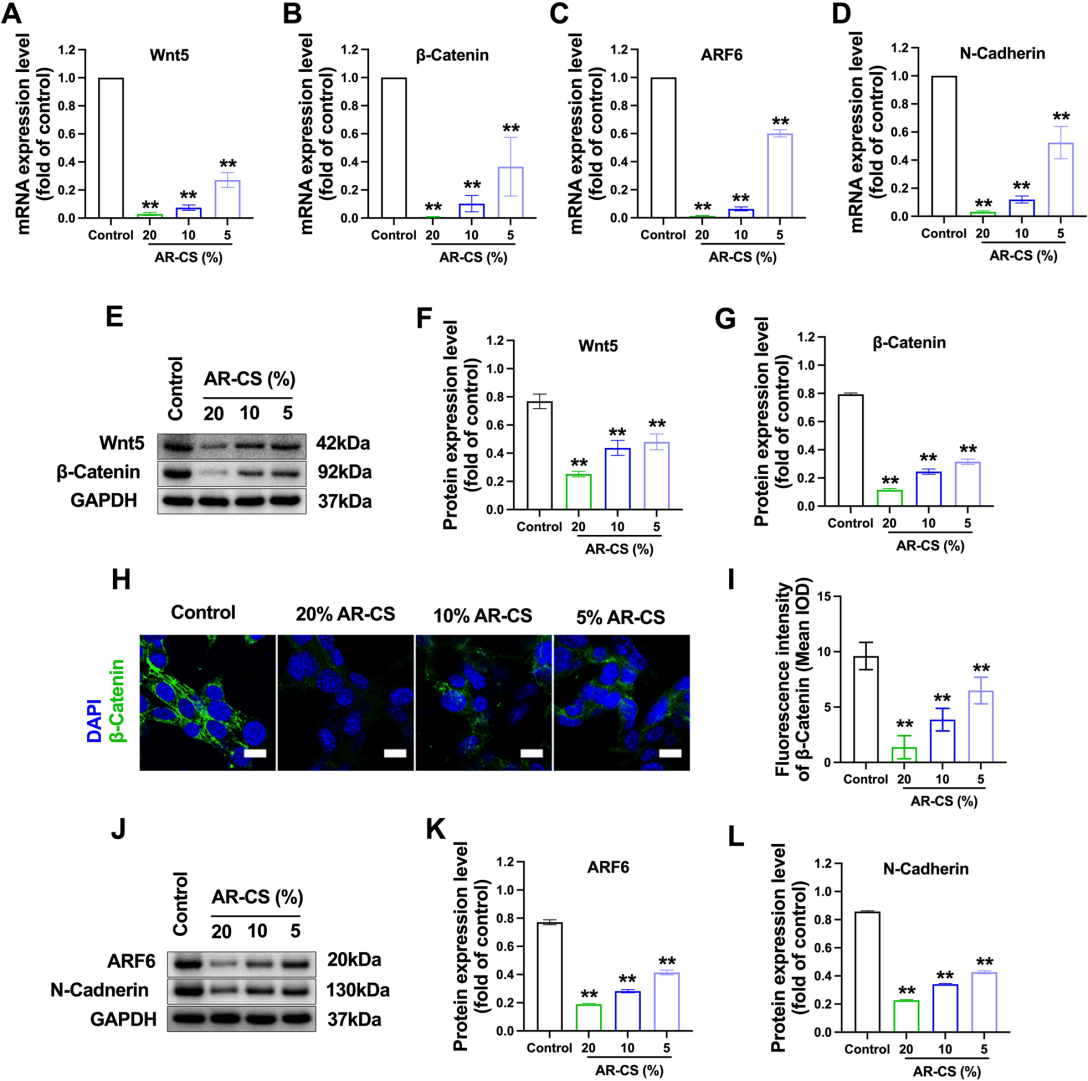
AR-CS inhibits Wnt/β-Catenin signaling and EMT of SW620 cells. Different concentrations of AR-CS (0%, 20%, 10%, 5%) treated SW620 cells for 24 h, real-time qPCR was used to detect Wnt5 (**A**), β-Catenin (**B**), ARF6 (**C**), and N-Cadherin (**D**) mRNA expression. Western blot was used to detect Wnt5 and β-Catenin protein expression (**E**), Wnt5 (**F**) and β-Catenin (**G**) protein expression level was normalized to control group. Immunofluorescent staining was used to detect β-Catenin expression (**H**) and the fluorescence density was calculated (**I**). Scale bar = 20 µm. Western blot was used to detect ARF6 and N-Cadherin protein expression (**J**), ARF6 (**K**) and N-Cadherin (**L**) protein expression level was normalized to control group. N = 6, compared with the control group, **p < 0.01

After observing that AR-CS has the ability to inhibit the proliferation and migration of colorectal cancer cells, we tried to find the possible mechanism behind this. We focus on the Wnt/β-Catenin signaling. After 20% / 10% / 5% gradient concentration of AR-CS acted on SW620 cells for 24 h, Wnt 5 and β-Catenin mRNA levels and protein levels in SW620 cells were significantly reduced **(**Fig. 3A, B, E–G). β-Catenin was retested using immunofluorescence, and the results showed AR-CS significantly reduced the fluorescence intensity of β-Catenin in SW620 cells **(**Fig. 3H, I). Furthermore, we detected the expression of ARF6 and N-Cadherin, which are related to tumor cell proliferation, adhesion and migration ability. After the application of AR-CS, the results showed that the 20%/10%/5% gradient concentration of AR-CS significantly reduced the mRNA level and protein level of ARF6 and N-Cadherin after 24 h in SW620 cells (Fig. 3C, D, J–L).

### Overexpression of Wnt 5 or β-Catenin eliminates the inhibition ability of AR-CS in proliferation and migration of SW620 cells

**Fig. 4:**
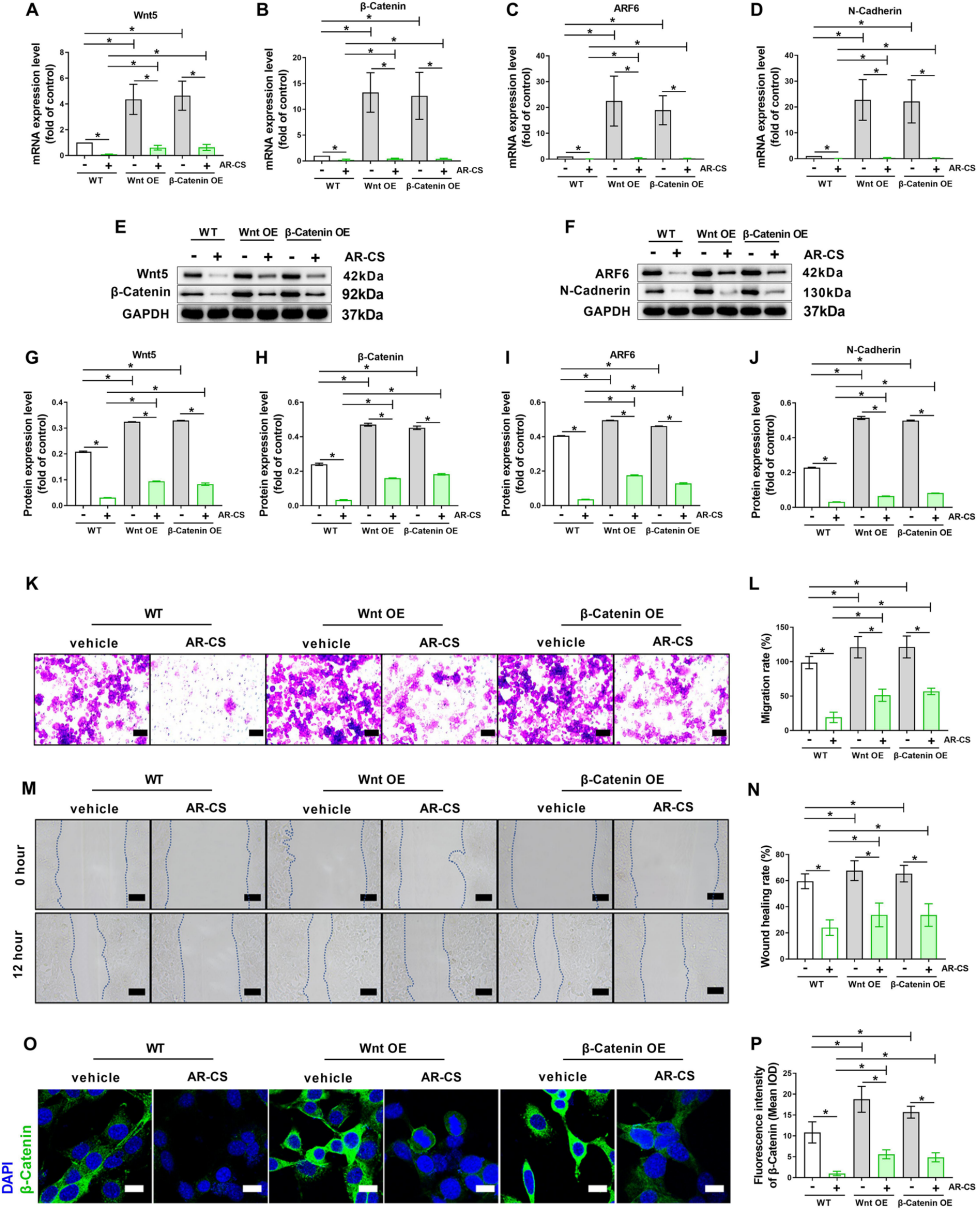
Overexpression of Wnt5 or β-Catenin eliminates the inhibition ability of AR-CS in proliferation and migration of SW620 cells. WT, Wnt5 over-expressed, and β-Catenin over-expressed SW620 cells with or without 20% AR-CS treated for 24 h, real-time qPCR was used to detect Wnt5 (**A**), β-Catenin (**B**), ARF6 (**C**), and N-Cadherin (**D**) mRNA expression. Western blot was used to detect Wnt5, β-Catenin (**E**), ARF6, and N-Cadherin (**F**) protein expression. Wnt5 (**G**), β-Catenin (**H**), ARF6 (**I**), and N-Cadherin (**J**) protein expression level was normalized to control group. Transwell assay (**K**) was used to detect cell migration rate (**L**). Wound scratch assay (**M**) was used to detect cell wound healing rate (**N**). Immunofluorescent staining was used to detect β-Catenin expression (**O**) and the fluorescence density was calculated (**P**). Scale bar = 100 µm in **K**, Scale bar = 400 µm in **M**, Scale bar = 20 µm in **O**. N = 6, in indicating comparison, *p < 0.05

To determine whether the inhibition of Wnt/β-Catenin signaling is the potential cause of AR-CS to inhibit the proliferation and migration of SW620 cells, the Wnt 5 or β-Catenin overexpression plasmids was used to temporarily increased the mRNA level of Wnt5 or β-Catenin in SW620 cells (Fig. 4A, B). The results of western blot showed that overexpression of Wnt 5 or β-Catenin promoted an increase in the protein level of Wnt 5 or β-Catenin in SW620 cells (Fig. 4E, G, H). Compared with WT-type SW620, overexpression of Wnt 5 or β-Catenin significantly increased the mRNA level and protein level of ARF6 and N-Cadherin (Fig. 4C, D, F, I, J), and also promoted the proliferation and migration of SW620 cells (Fig. 4K–N). The results of 20% AR-CS in the WT-type SW620 here repeatedly confirmed the inhibitory effect of AR-CS on the mRNA levels and protein levels of Wnt5, β-Catenin, ARF6 and N-Cadherin (Fig. 4A–J), and its inhibitory effect on the proliferation and migration of SW620 cells (Fig. 4K–N). Compared with WT-type SW620 cells, in cells with overexpression of Wnt5 or β-Catenin, the inhibitory effect of 20% AR-CS on the mRNA level and protein level of Wnt5, β-Catenin, ARF6 and N-Cadherin was partially reversal (Fig. 4A–J), and the inhibitory effect of AR-CS on the proliferation and migration of SW620 cells was greatly weakened (Fig. 4K–N). β-Catenin expression was retested using immunofluorescence, and the fluorescence intensity of β-Catenin showed consistent changes with western blot results in WT, Wnt5 over-expressed, and β-Catenin over-expressed SW620 cells with or without 20% AR-CS treatment (Fig. 4O, P).

### Knock-down of Wnt 5 or β-Catenin promotes the inhibition ability of AR-CS in proliferation and migration of SW620 cells

**Fig. 5:**
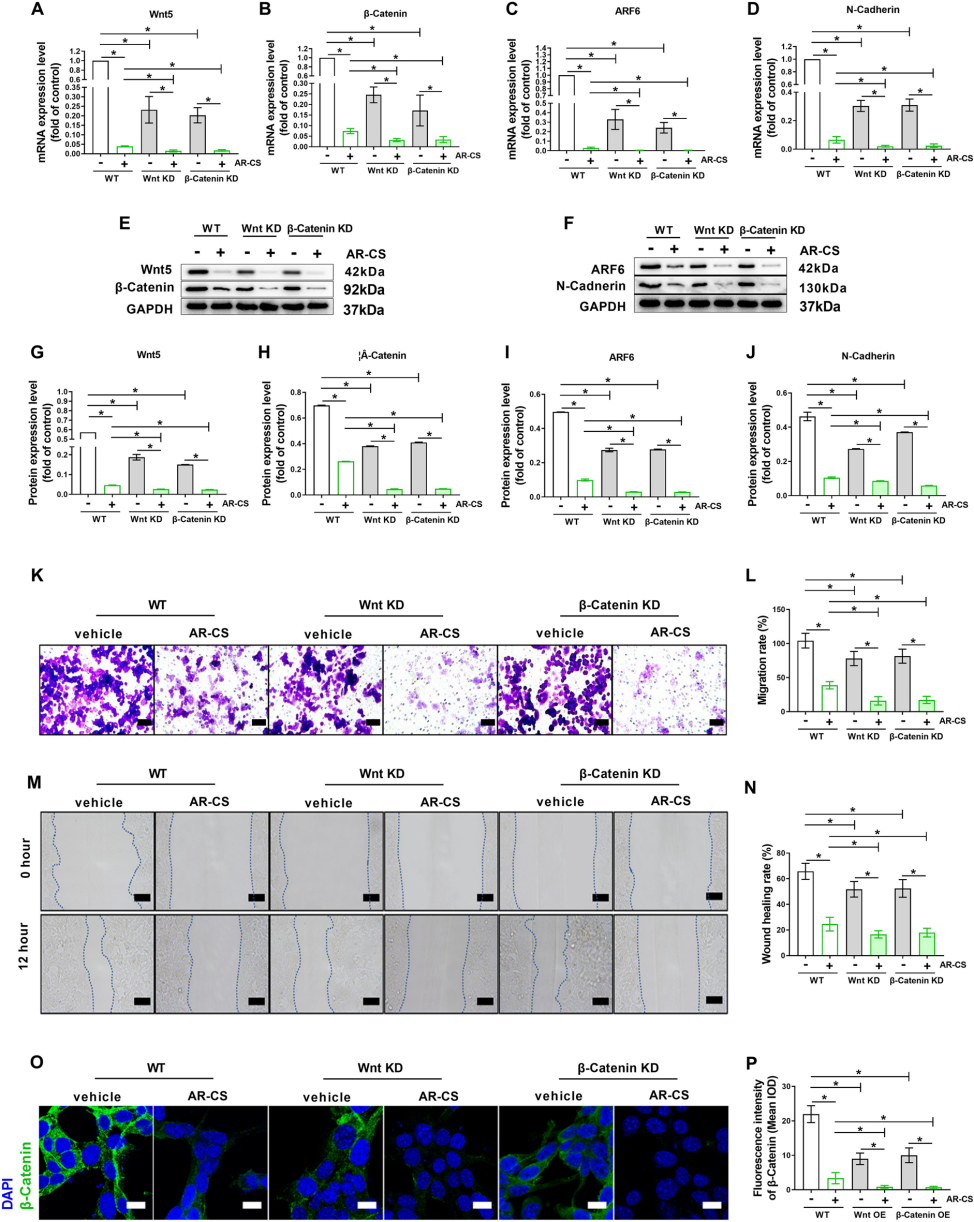
Knock-down of Wnt5 or β-Catenin promotes the inhibition ability of AR-CS in proliferation and migration of SW620 cells. WT, Wnt5 knock-down, and β-Catenin knock-down SW620 cells with or without 20% AR-CS treated for 24 h, real-time qPCR was used to detect Wnt5 (**A**), β-Catenin (**B**), ARF6 (**C**), and N-Cadherin (**D**) mRNA expression. Western blot was used to detect Wnt5, β-Catenin (**E**), ARF6, and N-Cadherin (**F**) protein expression. Wnt5 (**G**), β-Catenin (**H**), ARF6 (**I**), and N-Cadherin (**J**) protein expression level was normalized to control group. Transwell assay (**K**) was used to detect cell migration rate (**L**). Wound scratch assay (**M**) was used to detect cell wound healing rate (**N**). Immunofluorescent staining was used to detect β-Catenin expression (**O**) and the fluorescence density was calculated (**P**). Scale bar = 100 µm in **K**, Scale bar = 400 µm in **M**, Scale bar = 20 µm in **O**. N = 6, in indicating comparison, *p < 0.05

To further clarify the role of Wnt/β-Catenin signaling in AR-CS inhibiting the proliferation and migration of SW620 cells, Wnt 5 or β-Catenin siRNA was used to knock-down the mRNA level of Wnt 5 or β-Catenin in SW620 cells (Fig. 5A, B), Western blot results showed that knock-down of Wnt 5 or β-Catenin significantly reduced the protein level of Wnt 5 or β-Catenin in SW620 cells (Fig. 5E, G, H). Compared with WT-type SW620, knockdown of Wnt5 or β-Catenin significantly reduced the mRNA and protein levels of ARF6 and N-Cadherin in SW620 cells (Fig. 5C, D, F, I, J), while inhibiting the proliferation and migration of SW620 cells (Fig. 5K–N). The results of 20% AR-CS in the WT-type SW620 here repeatedly for the third time confirmed the inhibitory effect of AR-CS on the mRNA and protein levels of Wnt 5, β-Catenin, ARF6 and N-Cadherin (Fig. 5A–J), and its inhibitory effect on the proliferation and migration of SW620 cells (Fig. 5K–N). Compared with WT-type SW620 cells, in Wnt 5 or β-Catenin knockdown cells, the inhibitory effect of 20% AR-CS on the mRNA and protein levels of Wnt 5, β-Catenin, ARF6 and N-Cadherin was enhanced (Fig. 5A–J), and the inhibitory effect of AR-CS on the proliferation and migration of SW620 cell was enhanced (Fig. 5K–N). β-Catenin expression was retested using immunofluorescence, and the fluorescence intensity of β-Catenin showed consistent changes with western blot results in WT, Wnt5 knock-down, and β-Catenin knock-down SW620 cells with or without 20% AR-CS treatment (Fig. 5O, P).

### Wnt 5 or β-Catenin inhibitor promotes the inhibition ability of AR-CS in proliferation and migration of SW620 cells

**Fig. 6:**
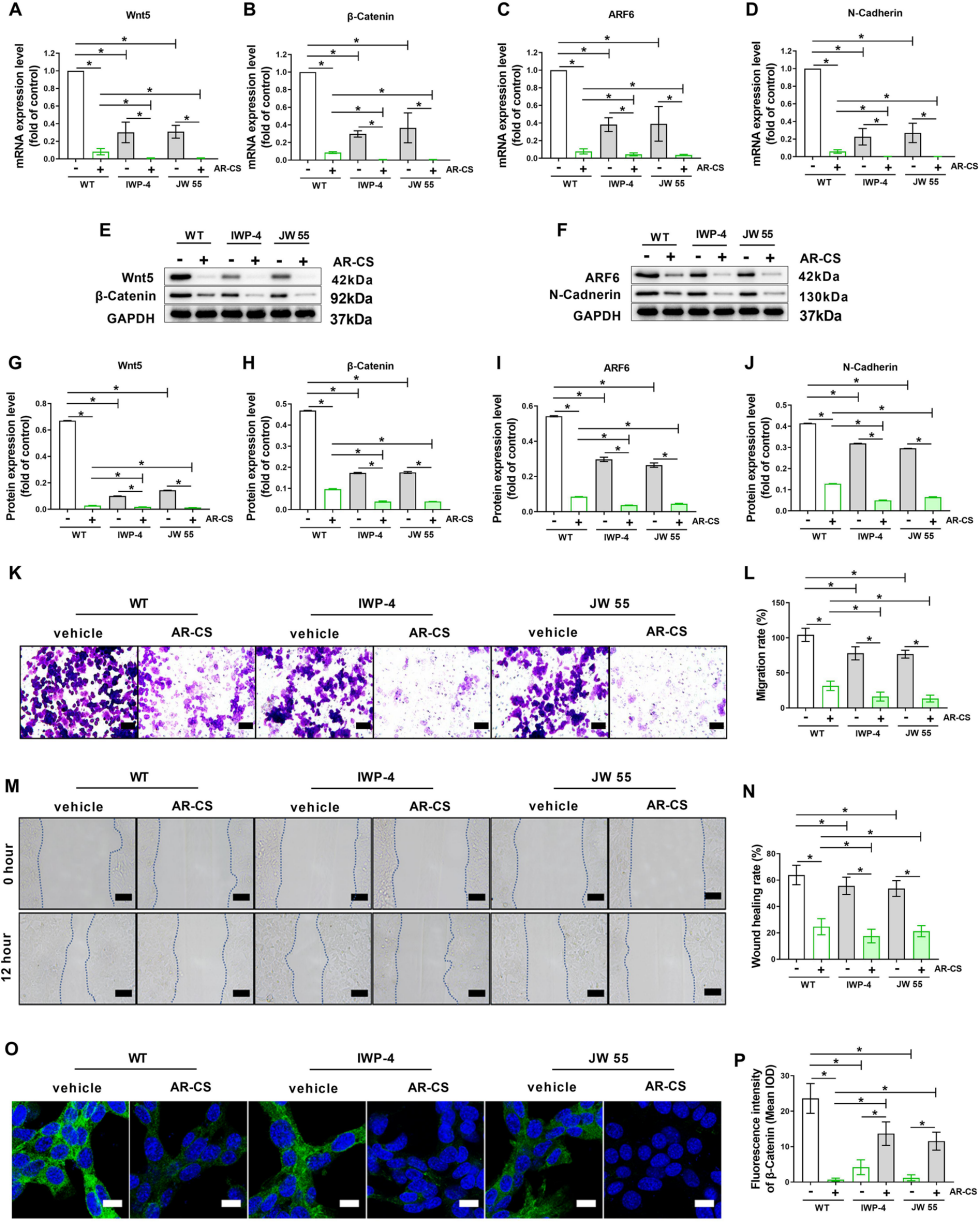
Wnt5 or β-Catenin inhibitor promotes the inhibition ability of AR-CS in proliferation and migration of SW620 cells. SW620 cells were treated with or without 20% AR-CS, Wnt5 inhibitor (IWP-4), and β-Catenin inhibitor (JW55) treated for 24 h, real-time qPCR was used to detect Wnt5 (**A**), β-Catenin (**B**), ARF6 (**C**), and N-Cadherin (**D**) mRNA expression. Western blot was used to detect Wnt5, β-Catenin (**E**), ARF6, and N-Cadherin (**F**) protein expression. Wnt5 (**G**), β-Catenin (**H**), ARF6 (**I**), and N-Cadherin (**J**) protein expression level was normalized to control group. Transwell assay (**K**) was used to detect cell migration rate (**L**). Wound scratch assay (**M**) was used to detect cell wound healing rate (**N**). Immunofluorescent staining was used to detect β-Catenin expression (**O**) and the fluorescence density was calculated (**P**). Scale bar = 100 µm in **K**, Scale bar = 400 µm in **M**, Scale bar = 20 µm in **O**. N = 6, in indicating comparison, *p < 0.05

Wnt 5 inhibitor (IWP-4), or β-Catenin inhibitor (JW55) alone or together with AR-CS verified the above results. After incubating SW620 cells with IWP-4 or JW55 for 24 h, the mRNA and protein levels of Wnt 5 or β-Catenin were significantly reduced. At the same time, the mRNA and protein levels of ARF6 and N-Cadherin, and the proliferation and migration of SW620 cells were also reduced (Fig. 6A–J). Futhermore, AR-CS incubated with IWP-4 or JW55 increased the mRNA and protein levels of Wnt 5, β-Catenin, ARF6 and N-Cadherin, compared with the AR-CS alone group (Fig. 6A–J). The inhibitory effect of AR-CS on the proliferation and migration of SW620 cells was also enhanced by Wnt 5 inhibitor (IWP-4), or β-Catenin inhibitor (JW55) (Fig. 6K–N). β-Catenin expression was retested using immunofluorescence, and the fluorescence intensity of β-Catenin showed consistent changes with western blot results in SW620 cells with or without 20% AR-CS, Wnt5 inhibitor (IWP-4), and β-Catenin inhibitor (JW55) treatment (Fig. 6O, P).

### AR decoction inhibits tumor growth in nude mouse xenograft model

**Fig. 7:**
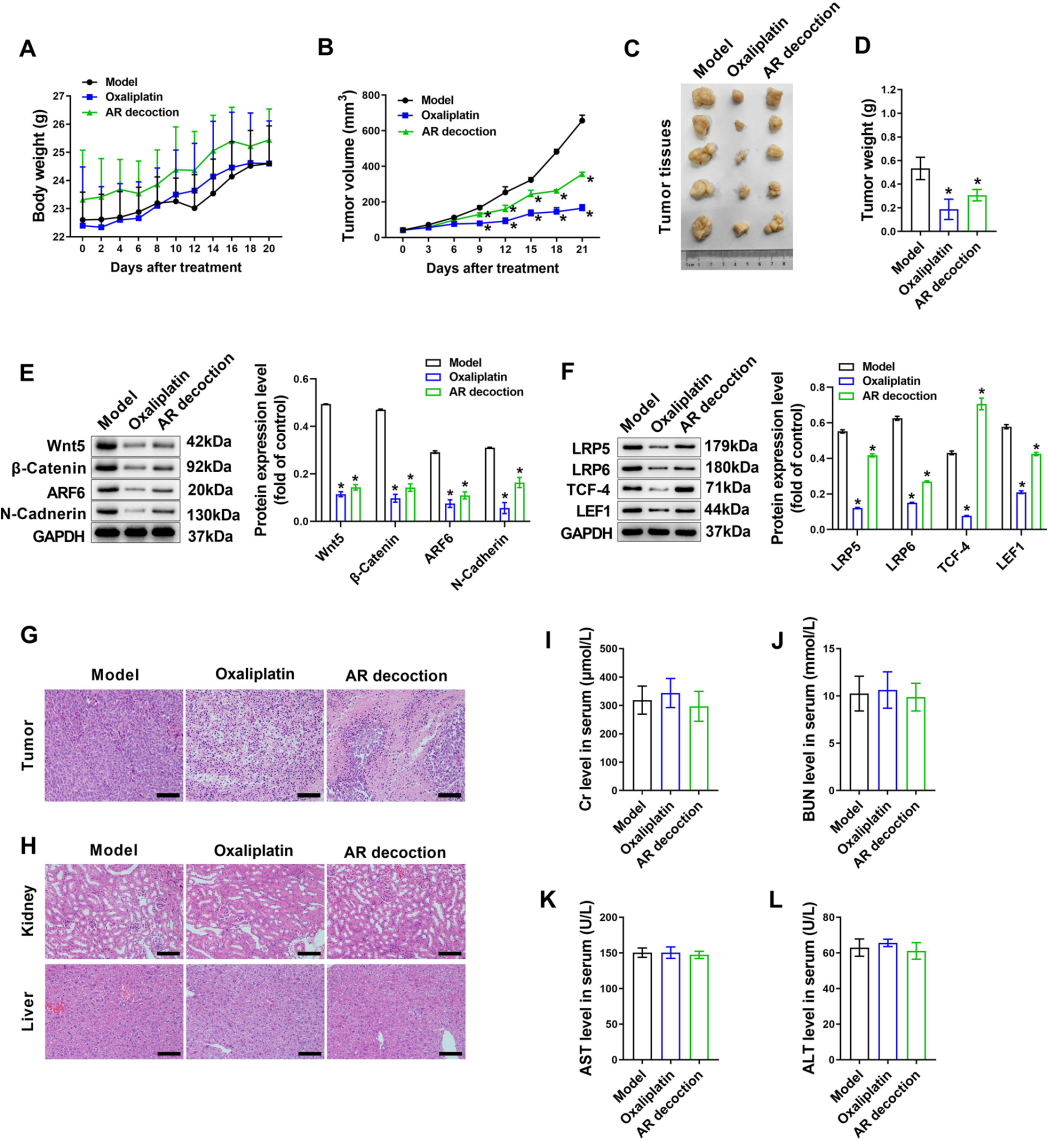
AR decoction inhibits tumor growth in nude mouse xenograft model. Intragastric administration of AR decoction (6.5 g/kg) for a nude mouse xenograft model was performed for 21 days. Oxaliplatin was used as a positive drug. The body weight of mice was weighed every two days (**A**). The tumor volume of mice was measured and calculated every three days (**B**). The tumors of mice (**C**) were taken and weighed (**D**) after administration of AR decoction. Western blot was used to detect Wnt5, β-Catenin, ARF6, N-Cadherin (**E**), LRP5, LRP6, TCF-4, and LEF1 (**F**) protein expression in tumors, the protein expression level was normalized to model group. HE staining was used to observe changes of organizational morphology in tumor (**G**), liver and kidney (**H**). Scale bar = 200 µm in **G** and **H**. Blood biochemical test was used to dectet Cr (**I**), BUN (**J**), AST (**K**), and ALT (**L**) levels in serum in mice. N = 5, compared with the model group, *p < 0.05

After confirming that AR-CS can reduce proliferation and migration of SW620 cells by inhibiting Wnt5/β-Catenin, we further verified the effect of AR decoction on tumor growth in vivo. Nude mice were injected subcutaneously with SW620 cells to replicate xenograft tumor mouse models, and AR decoction was given daily for 21 days after inoculation. Continuous weight data showed that neither AR decoction nor the positive control drug oxaliplatin had a significant effect on the body weight of xenograft mice (Fig. 7A). Continuous measurement of tumor volume showed that AR decoction or oxaliplatin significantly reduced the tumor volume of xenograft mice from 9 days treatment (Fig. 7B). In model mice treated with AR decoction or Oxaliplatin, the weight of the tumor was significantly reduced (Fig. 7C, D), and the protein levels of Wnt 5, β-Catenin, ARF6 and N-Cadherin in the tumor were significantly down-regulated (Fig. 7E). Moreover, the protein levels of LRP5, LRP6, TCF-4, and LEF1 in the tumor were also significantly down-regulated by AR decoction or oxaliplatin treatment in xenograft mice (Fig. 7F). In the HE section of the tumor, it can be seen that AR decoction or Oxaliplatin treatment caused cell apoptosis in the tumor tissue (Fig. 7G). The results of HE staining of the kidney and liver showed that AR decoction or Oxaliplatin treatment did not cause pathological damage to the kidney and liver (Fig. 7H). Blood biochemistry tests showed that AR decoction or oxaliplatin had no influence in serum Cr, BUN, AST, and ALT in xenograft mice (Fig. 7I–L).

## Discussion

Here we report a traditional Chinese medicine AR decoction to treat colorectal cancer by inhibiting Wnt5/β-Catenin signaling. In fact, traditional Chinese medicine have been used in the treatment of clinical tumors for many years [27], not only colon cancer [28], but also a variety of cancers, including breast cancer [29], glioblastoma [30], and pancreatic cancer [31]. Arsenic trioxide derived from traditional medicine has achieved exciting results in the treatment of leukemia [32]. The AR decoction explored in our current research has been used in clinical practice in China to treat CRC, but the unclear mechanism limits its clinical use. In the current study, we have provided evidence of AR decoction in the treatment of CRC in vitro and in vivo, and confirmed that Wnt 5/β-Catenin signaling plays an important role in this process.

The Wnt protein family plays a key role in tumor development through the classic Wnt/β-catenin pathway, or the non-canonical pathway independent of β-catenin [33]. The Wnt signaling pathway is composed of functional proteins, enzymes, transcriptional regulatory factors, etc. Among them, β-catenin is a multifunctional protein involved in cell adhesion and signal transduction, and is a key component of the Wnt signaling pathway [34]. When the Wnt/β-catenin pathway is activated, the Wnt ligand-related protein interacts with the receptor, resulting in the failure of the β-catenin polyprotein complex to form, and the increasing of free β-catenin protein level in the cytoplasm, then excessive β-Catenin enters the nucleus and interacts with nuclear transcription factors to initiate the transcription of Wnt pathway target genes and the expression of related proteins, including VEGF/E-cadherin/Cyclin-D1 [35], which leads to uncontrolled cell proliferation and decreased adhesion, angiogenesis, causing malignant proliferation, invasion and metastasis of tumor cells [36]. Study showed that Huangqi decoction improved renal tubulointerstitial fibrosis in mice by inhibiting Wnt/β-catenin signaling pathway [37], and the extract of Huangqi and Ezhu inhibited the growth of Lewis lung cancer cells in xenograft mouse models by suppressing MAPK and VEGF signals [38]. Our present study showed AR decoction could inhibit tumor growth in mouse models of CRC xenograft tumors via inhibiting Wnt/β-catenin signaling.

In the current study, first, we used AR-CS to interfere with SW620 cell line. After obtaining the appropriate intervention concentration and intervention time, it was confirmed that AR-CS could significantly inhibit the proliferation and migration of SW620 cells. We also confirmed that AR-CS inhibited the expression of Wnt 5 and β-catenin in SW620 cells in a concentration-dependent manner. In SW620 cells overexpressing Wnt5 or β-catenin, the inhibitory effect of AR-CS on the proliferation and migration of SW620 cells disappeared. Our research also showed that in SW620 cells, either knockdown of Wnt5 alone, β-catenin alone, or use of Wnt5 or β-catenin inhibitors directly inhibited the proliferation and migration of SW620 cells. As previously reported, inhibition of Wnt5/β-catenin is an effective treatment for CRC [20, 39]. When AR-CS was applied to Wnt 5 or β-catenin knock-down SW620 cells, or when AR-CS was used in combination with Wnt 5 or β-catenin inhibitors, the inhibitory effect of AR-CS on the proliferation and migration of SW620 cells was significantly enhanced, compared with WT-type SW620 or compared with SW620 cells treated with AR-CS alone. In summary, these results provide evidence that AR decoction inhibited the proliferation and migration of colorectal cancer cells by inhibiting Wnt5/β-catenin.

In the process of AR-CS reducing the proliferation and migration of SW620 cells by inhibiting Wnt5/β-catenin, our data also showed that the expression of ARF6 and N-Cadnerin in SW620 cells was significantly reduced by AR-CS. The small GTPase protein ARF6 has been proved to play a non-negligible role in promoting the proliferation, invasion and migration of a variety of tumor cells. By stimulating invasion ability, disrupting E-cadherin-mediated cell adhesion, and inducing the recycling of β-catenin, the high expression of ARF6 promotes the development of breast cancer, renal cell carcinoma, lung adenocarcinoma and colorectal cancer [40–42]. N-cadherin is high expression in many tumors, including colorectal cancer [43]. Normal epithelial cells express E-cadherin, a cell adhesion molecule closely related to N-cadherin [44]. Poorly differentiated cancer cells often no longer express E-cadherin, but show high expression of N-cadherin. A large number of studies have shown that the absence of E-cadherin and the abnormally high expression of N-cadherin can cause tumor cells to lose polarity, resist apoptosis, and increase invasiveness and metastasis [45]. Inhibition of N-cadherin has the potential to induce tumor cell apoptosis and inhibit metastasis [46].

When we applied AR-CS on SW620 cells overexpressing Wnt5 or β-catenin, the inhibitory effect of AR-CS on the expression of ARF6 and N-Cadnerin disappeared, indicating that the effect of AR-CS depended on Wnt5/β-catenin signal. In SW620 cells, either knock-down of Wnt 5 alone, β-catenin alone, or use of Wnt5 or β-catenin inhibitors directly inhibited the expression of ARF6 and N-Cadnerin in SW620. And when we use AR-CS on Wnt 5 or β-catenin knock-down SW620 cells, or when AR-CS was combined with Wnt 5 or β-catenin inhibitors, the inhibitory effect of AR-CS on the expression of ARF6 and N-Cadnerin in SW620 cells was enhanced, compared with wild-type SW620 cells or compared with SW620 cells treated with AR-CS alone. Overall, the current results provide evidence that AR decoction inhibits the expression of ARF6 and N-Cadnerin in colorectal cancer cells by inhibiting Wnt5/β-catenin.

In this study, we also provided evidence that AR decoction inhibited tumor proliferation in CRC mice, and inhibited Wnt5/β-catenin signaling and the expression of ARF6 and N-Cadnerin in tumors of CRC mice. More importantly, in vivo we performed protein expression detections of LRP5 and LRP6 which were associated with Wnt assembly [47], and protein expression detections of transcription factor TCF-4 and LEF1 which were regulated by β -catenin [6]. We confirmed that AR inhibits LRP5, LRP6, TCF-4, and LEF1 expression in vivo.

Some deficiencies of this research are still worthy of in-depth exploration, including the failure to explain in depth how AR decoction interacts with Wnt5/β-catenin signals to treat CRC; and the failure to collect solid tumor samples from CRC patients receiving AR decoction treatment in China to verify the changes in Wnt5/β-catenin signal and the expression of ARF6 and N-Cadnerin in patients. Some studies have shown that Huangqi or Ezhu could promote the efficacy of chemotherapeutic drugs or reduce drug resistance in cancer treatment [48, 49]. Whether AR decoction has these effects is another interesting line of research.

## Conclusion

The present study provided evidence that AR decoction inhibits Wnt5/β-catenin signaling and inhibits the development of CRC in vitro and in vivo. AR decoction is a promising traditional medicine in the clinical treatment of CRC.

## Data Availability

Data will be available from the authors upon reasonable request and with permission of Yong Bian after submission of the thesis.
